# Epigenetic Regulation of the Vascular Endothelium by Angiogenic LncRNAs

**DOI:** 10.3389/fgene.2021.668313

**Published:** 2021-08-26

**Authors:** Noeline Subramaniam, Ranju Nair, Philip A. Marsden

**Affiliations:** ^1^Marsden Lab, Institute of Medical Sciences, University of Toronto, Toronto, ON, Canada; ^2^Marsden Lab, Keenan Research Centre in the Li Ka Shing Knowledge Institute, St. Michael’s Hospital, Toronto, ON, Canada; ^3^Marsden Lab, Department of Laboratory Medicine and Pathobiology, University of Toronto, Toronto, ON, Canada; ^4^Department of Medicine, University of Toronto, Toronto, ON, Canada

**Keywords:** endothelial cell, lncRNA, angiogenesis, epigenetics, gene regulation, vascular

## Abstract

The functional properties of the vascular endothelium are diverse and heterogeneous between vascular beds. This is especially evident when new blood vessels develop from a pre-existing closed cardiovascular system, a process termed angiogenesis. Endothelial cells are key drivers of angiogenesis as they undergo a highly choreographed cascade of events that has both exogenous (e.g., hypoxia and VEGF) and endogenous regulatory inputs. Not surprisingly, angiogenesis is critical in health and disease. Diverse therapeutics target proteins involved in coordinating angiogenesis with varying degrees of efficacy. It is of great interest that recent work on non-coding RNAs, especially long non-coding RNAs (lncRNAs), indicates that they are also important regulators of the gene expression paradigms that underpin this cellular cascade. The protean effects of lncRNAs are dependent, in part, on their subcellular localization. For instance, lncRNAs enriched in the nucleus can act as epigenetic modifiers of gene expression in the vascular endothelium. Of great interest to genetic disease, they are undergoing rapid evolution and show extensive inter- and intra-species heterogeneity. In this review, we describe endothelial-enriched lncRNAs that have robust effects in angiogenesis.

## Introduction

The cardiovascular system is a complex and dynamic network of blood vessels pumping blood from the heart to the rest of the body. Through the blood vessels, nutrients and oxygen are delivered to the cells, and carbon dioxide and waste products are removed. This occurs at a rapid rate in advanced species, and to maintain this, there is a tight interplay of multiple hemodynamic forces including circumferential stretch, hydrostatic pressure, shear stress and rates of blood flow. At the interface between the circulating blood and the vascular wall is the vascular endothelium, acting as a dynamic barrier. The endothelium is a monolayer of endothelial cells (ECs) that lines the entire closed cardiovascular system. We have previously argued that ECs are professional sensors of hemodynamic forces (Ku et al., 2019). ECs sense and respond to these forces, which in turn affect EC phenotype.

A finite number of cis-DNA elements and associated trans-factors mediate the nuclear-based response to the interplay of these varied hemodynamic factors. One such cis-DNA element that is activated by atheroprotective, laminar flow is the shear stress response element (SSRE), which was first identified in the promoter region of platelet-derived growth factor-B (PDGF-B) (Resnick et al., 1993). We now know that the SSRE is detected in many other flow-regulated EC genes such as intercellular adhesion molecule 1 (ICAM1), endothelin-1 (ET-1/EDN1), monocyte chemoattractant protein 1 (MCP1)/chemokine (C-C motif) ligand 2 (CCL2), and the prototypic EC gene responsible for nitric oxide production, endothelial nitric oxide synthase (eNOS) (Ku et al., 2021). Another important flow-regulated cis-DNA element is Krüppel-like factor (KLF). The KLFs are zinc-finger transcription factors (Anderson et al., 1995). In particular, it is known that KLF2 and KLF4 are important flow-regulated transcription factors that signal through the MEF5/ERK5/MEF2 pathway to mediate transcription of many flow-responsive genes (Dekker et al., 2002b; Parmar et al., 2005; Ohnesorge et al., 2010; Le et al., 2013; Sangwung et al., 2017). KLF2 regulates vascular tone by inducing expression of eNOS (Dekker et al., 2005; Parmar et al., 2005). Models of atherosclerosis confirm the critical role for these KLF transcription factors in vascular homeostasis. In apolipoprotein E (ApoE) deficient mice with hemizygous KLF2 deficiency, there is a notable increase in atherosclerosis (Atkins et al., 2008). Similar results are observed with EC-specific loss of KLF4 in ApoE deficient mice (Zhou et al., 2012). Together, these studies demonstrate an important atheroprotective role for KLF2 and KLF4. Finally, there is the myocyte enhancer factor-2 (MEF2) family, members of which bind to the promoter region of KLF2 and regulate its expression under shear stress (Kato et al., 1997; Parmar et al., 2005; Wang et al., 2010). EC-specific deletions in mice of MEF2 factors, Mef2a, -c, and -d, disrupt vascular homeostasis (Lu et al., 2021). Combined deletion of these MEF2 factors significantly decreased KLF2/KLF4 expression. In summary, these independent cis-DNA elements, namely the SSRE, KLF and MEF2 elements mediate transcriptional responses to changes in shear stress.

Adding to this classic cis-trans paradigm, EC gene expression is also regulated by epigenetic mechanisms. Broadly defined, epigenetics refers to chromatin-based mechanisms important in the regulation of gene expression that do not involve changes to the DNA sequence *per se* (Matouk and Marsden, 2008; Yan et al., 2010; Webster et al., 2013). Epigenetic mechanisms include DNA methylation, histone modifications and RNA-based mechanisms, including long non-coding RNAs (lncRNAs). They are highly responsive to changes in the environment, making them quite dynamic. Epigenetic mechanisms have profound effects on many biological processes in which ECs participate in, especially hemodynamic regulation and angiogenesis. Short non-coding RNAs, such as microRNAs (miRNAs), have been gaining scientists’ attention for their post-transcriptional effects since the early 2000s (Battistella and Marsden, 2015). In recent years, a newer class of non-coding RNAs called long non-coding RNAs have emerged, and they have been shown to be important regulators of gene expression in health and disease. LncRNAs tend to be enriched in the nucleus, where they can act as epigenetic modifiers of gene expression (Man and Marsden, 2019). Some of the best studied lncRNAs are X-inactive specific transcript (XIST) which is known to inactivate one of the X chromosomes in females; HOX transcript antisense RNA (HOTAIR), which is involved in limb development; and antisense non-coding RNA in the Inhibitors of CDK4 (INK4) locus (ANRIL), which is strongly correlated with cardiovascular disease risk. The identification and characterization of angiogenic lncRNAs has introduced the idea that lncRNAs may serve as biomarkers and/or therapeutic targets for diseases in which angiogenesis is disrupted, such as in cancers or cardiovascular disease. In this review, we will discuss how nuclear endothelial-enriched lncRNAs, affect EC angiogenesis. We will especially highlight the STEEL, GATA6-AS and MANTIS lncRNAs.

## Long Non-Coding RNAs

Historically believed to be “transcriptional noise” or “dark matter,” lncRNAs have emerged as key modulators of many biological processes. Scientists have identified thousands of lncRNAs, with the online database “LncBook” citing > 270,000 lncRNAs in humans (Ma et al., 2019). However, the number of lncRNAs that have been functionally characterized is ∼<1% of those identified (Quek et al., 2015). LncRNAs are primarily characterized by their length as >200 nucleotides long, mainly to distinguish this class of non-coding molecules from shorter transcripts (Mercer et al., 2009; Mattick and Rinn, 2015). LncRNAs can be 5′-capped, spliced, polyadenylated, and often have low expression levels relative to protein-coding genes (Guttman et al., 2009; Derrien et al., 2012). As their name suggests, they are not translated into proteins and thus, often have trivial or non-functional open reading frames (ORFs). This can be assessed through bioinformatic analysis of coding domain sequence, secondary structure, di/tri-nucleotide sequence frequencies and cross-species conservation (Ulitsky and Bartel, 2013; Ventola et al., 2017). LncRNAs can be classified based on several criteria, but broadly are often grouped by their organization relative to other genes, due to a lack of clarity on their sequence-structure-function relationship. They can be described as intronic, intergenic, antisense, bidirectional, enhancer, or promoter-associated lncRNAs.

**FIGURE 1:**
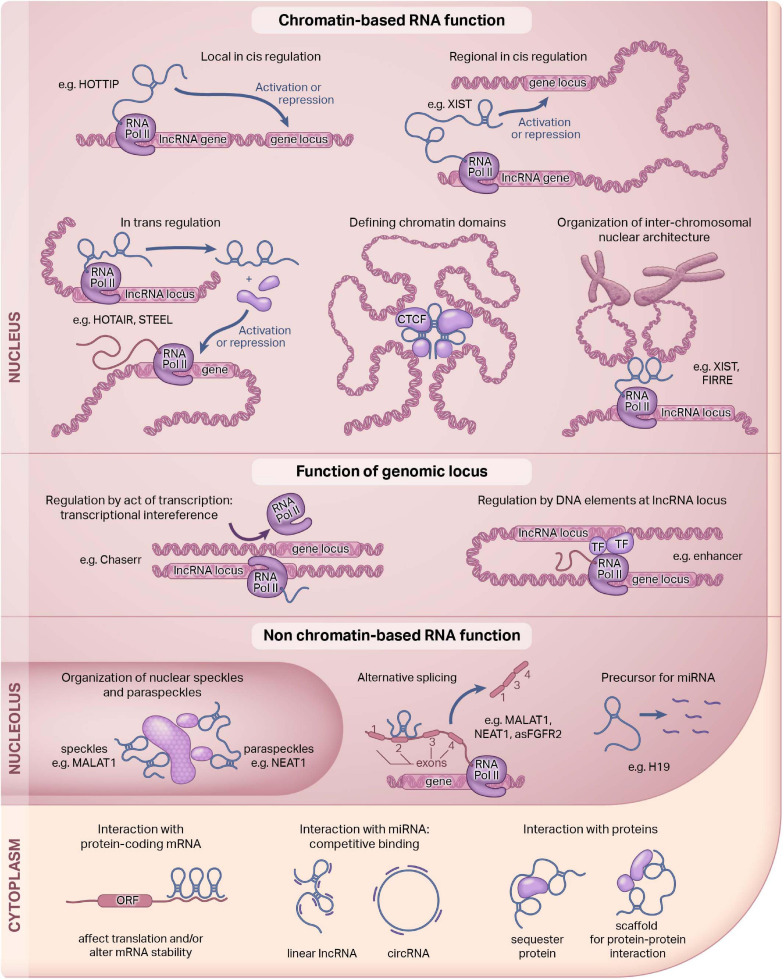
Functions of long non-coding RNAs. Long non-coding RNAs (lncRNAs) are a diverse class of molecules that are distributed throughout the cell. The function of lncRNAs are dependent, in part, on their subcellular localization. Here, we illustrate known lncRNA functions in the nucleus and the cytoplasm. Our review focuses on the nuclear function of lncRNAs. This figure was adapted; it was originally published in Current Opinion in Pharmacology, Volume 45, Hon-Sum Jeffrey Man and Philip A Marsden, LncRNAs and epigenetic regulation of vascular endothelium: genome positioning system and regulators of chromatin modifiers, pp. 72–80, Copyright Elsevier, 2019.

LncRNAs can be present in the nucleus, cytoplasm, or mitochondria, and they may also be secreted (Rackham et al., 2011; van Heesch et al., 2014; see Figure 1). A lncRNA can also be expressed in multiple compartments, such as GAS5, which is expressed in both the nucleus and the cytoplasm (Kino et al., 2010). Since subcellular localization often confers function, the mechanism of action of a lncRNA can be inferred, in part, by defining where they are targeted (Cai and Cullen, 2007; Quinodoz and Guttman, 2014; Romero-Barrios et al., 2018; Hou et al., 2019; Mishra and Kanduri, 2019; Rom et al., 2019). Nuclear lncRNAs like XIST or HOTAIR are often important mediators of regulating epigenetic mechanisms (Mercer et al., 2009). They can act in *cis* or in *trans* by interacting with neighboring or non-neighboring genes to exert their effects (Rinn et al., 2007; Wang et al., 2011; Engreitz et al., 2013; Novikova et al., 2013). Cytoplasmic lncRNAs like Tie1-AS can interact with protein-coding genes, and others still can act as a scaffold for protein-protein interactions (Li et al., 2010). Many lncRNAs, including ones we will highlight in this review regulate gene expression through chromatin-based mechanisms.

Finally, non-coding RNAs can be enriched in particular cell- or tissue-types. For example, spliced-transcript endothelial-enriched lncRNA (STEEL) is an endothelial-enriched lncRNA that has EC-specific functions (Man et al., 2018). In contrast, there are lncRNAs like metastasis-associated lung adenocarcinoma transcript 1 (MALAT1) that is expressed at very high levels and is found widely across almost all cell types. Some lncRNAs are highly upregulated by environmental stimuli that are especially relevant to ECs, such as the lncRNA that enhances eNOS expression (LEENE), which is induced under physiological blood flow and pulsatile shear stress (Miao et al., 2018). Another example is GATA6-AS, which is hypoxia-responsive (Neumann et al., 2018). Disease-associated lncRNAs have also been identified, with the best example being ANRIL (McPherson et al., 2007; Holdt et al., 2010; Congrains et al., 2012). Through genome-wide association studies (GWAS), exons 13-19 of ANRIL comprise a disease-associated haplotype noted for a marked increased risk of coronary artery disease (CAD). Now we know that it is also associated with other cardiovascular diseases, such as ischemic peripheral vascular disease and ischemic stroke (Zeggini et al., 2007; Foroud et al., 2012; Kremer et al., 2015; Kong et al., 2016; Tan et al., 2019). Evidently, lncRNAs are biologically important and functionally diverse.

### How to Detect lncRNAs

There are many methods to identify lncRNAs, but microarrays were by far the method of choice for a long time—until the advent of deep RNA-sequencing (RNA-seq; Table 1; Mockler et al., 2005). While microarrays are high throughput, cost-effective, and computationally manageable, they also limit novel lncRNA discovery due to pre-determined probe sets; optimal probe coverage and density; and background noise from cross-hybridizations or weak binding (Uchida, 2017). Deep RNA-seq has become the current method of choice because it enables researchers to discover non-annotated transcripts, single nucleotide variations (SNVs), splice variants, novel splice junctions and gene fusion events (Sultan et al., 2008; Trapnell et al., 2009; Edgren et al., 2011; Djebali et al., 2012; Quinn et al., 2013). Moreover, deep RNA-seq has greater specificity and sensitivity enabling detection of low expression and rare transcripts as well as cell- and tissue-specific lncRNAs (Wang et al., 2014; Liu et al., 2015; Li et al., 2016). Thus, an important parameter of RNA-seq for accurate quantification is read depth. For RNAs of moderate abundance, ∼30–40 million reads are needed whereas for higher coverage (e.g., detecting rare and lowly expressed transcripts), reads of up to 500 million are recommended (Fu et al., 2014).

**TABLE 1:** Methods to detect a lncRNA.

Method	Function	Advantages	Disadvantages	Example lncRNA
Microarrays	To detect RNAs	High throughput; computationally manageable	Background noise from cross-hybridizations or weak binding	HOTAIR, STEEL (Rinn et al., 2007; Man et al., 2018)
RNA sequencing	To detect RNAs	Able to detect new transcripts; high sensitivity with increased read depth	LncRNA discovery limited by annotations and genome build accuracy	GATA6-AS (Neumann et al., 2018)

RNA-seq and next generation sequencing (NGS) has expanded significantly and over 400 methods have been established over the last decade (Hadfield and Retief, 2018). Emerging approaches include single-cell RNA-seq (scRNA-seq) which examines gene expression at a single-cell resolution, or assay for transposase-accessible chromatin sequencing (ATAC-seq) which locates regions of open chromatin, in genomic regions devoid of protein-coding genes. However, RNA-seq is not without its challenges. The analyses are more difficult and require more computational power; it is more costly; and multiple cycles of polymerase chain reaction (PCR) may introduce some amplification bias (Uchida, 2017). Importantly, lncRNA discovery is limited by annotations and genome build accuracy.

LncRNAs are difficult to annotate because of their low expression levels, our limited understanding of their sequence-function relationship and their lack of evolutionary conservation. Thus, lncRNAs are currently annotated primarily based on transcriptomic evidence (Uszczynska-Ratajczak et al., 2018). The 2 main annotation approaches are automated or manual. With manual annotation, humans strategically put together transcriptomic and genomic data to build models that can create relatively accurate annotations. Automated annotation uses transcriptome assembly approaches that are quick and not costly, but typically result in incomplete and inaccurate annotations. To date, manual annotations are more accurate. Moreover, manual annotations have a higher quality assessment of lncRNA coding potential (via mass spectrometry, PhyloCSF, UniProt, and Pfam) (Sonnhammer et al., 1998; Apweiler et al., 2004; Lin et al., 2011). The most widely used manual annotation is GENCODE, followed by Reference Sequence (Refseq) (Harrow et al., 2012; Pruitt et al., 2014; Fang et al., 2018). There has been a historical bias toward using cell lines, adult tissues and tumor samples to build these reference databases. LncRNAs specifically expressed in rare cell populations, in response to various environmental stimuli, and in development may be excluded from these annotations. As technologies continue to advance, we predict that these databases will become more comprehensive with higher confidence lncRNA annotations.

### How to Study lncRNAs

In order to study lncRNAs and their epigenetic and non-epigenetic functions, an arsenal of molecular biology techniques are employed by scientists. In Table 2, we outline the most commonly used methodologies to study lncRNA-chromatin and lncRNA-protein interactions. To identify chromatin associated lncRNAs, approaches have been broadly classified into either “one-to-many” or “all-to-all.” One-to-many approaches include chromatin isolation by RNA purification (ChIRP), capture hybridization analysis of RNA targets (CHART) and RNA antisense purification (RAP) (Chu et al., 2011; Simon et al., 2011; Engreitz et al., 2013). These methods are based on hybridizations of biotin-labeled probes targeted to lncRNAs of interest, followed by pull-down of the associated chromatin fraction. These techniques are limited by background noise from non-specific binding. RAP has relatively less background noise due to the use of longer probes, but these capture approaches could all be improved with further background corrections (Li and Fu, 2019). ChIRP, CHART, and RAP can be combined with other techniques to discover more interactions within a single experiment. One example is domain-specific ChIRP (dsChIRP), a method by which lncRNAs domain-by-domain are assessed to identify functional elements (Quinn et al., 2014). Deep sequencing and/or mass spectrometry can be also be combined to obtain more high-resolution data on lncRNA interactions.

**TABLE 2:** Methods to study lncRNA function.

Method	Function	Advantages	Disadvantages	LncRNA example	Method Reference
Chromatin Isolation by RNA purification (ChIRP)	To identify lncRNA-chromatin interactions	Probes can be designed without knowledge of structure or functional domains of target lncRNA	Background noise from non-specific binding	MEG3, HOTAIR (Chu et al., 2011; Iyer et al., 2017)	Chu et al., 2011
Capture hybridization analysis of RNA targets (CHART)	To identify lncRNA-chromatin interactions	Probes can be designed without knowledge of structure or functional domains of target lncRNA	Background noise from non-specific binding	NEAT1, MALAT1 (West et al., 2014)	Simon et al., 2011
RNA antisense purification (RAP)	To identify lncRNA-chromatin interactions	Longer probes mitigate background noise	Need probes to overlap entire length of lncRNA for capture	XIST, FIRRE (Engreitz et al., 2013; Hacisuleyman et al., 2014)	Engreitz et al., 2013
Mapping RNA-genome interactions (MARGI)	To identify lncRNA-chromatin interactions	Identifies native RNA-chromatin interactions *in vivo* and *in vitro*	Moderate sensitivity might decrease detection of low abundance chromatin-associated RNAs	XIST, SNHG1, NEAT1, MALAT1 (Sridhar et al., 2017)	Sridhar et al., 2017
Global RNA interaction with DNA sequencing (GRID-seq)	To identify lncRNA-chromatin interactions	Identifies genome-wide lncRNA-chromatin interactions *in situ*	Moderate sensitivity might decrease detection of low abundance chromatin-associated RNAs	MALAT1, NEAT1 (Li X. et al., 2017)	Li X. et al., 2017
Chromatin-associated RNA sequencing (ChAR-seq)	To identify lncRNA-chromatin interactions	Identifies genome-wide lncRNA-chromatin interactions *in situ*	Moderate sensitivity might decrease detection of low abundance chromatin-associated RNAs	*roX1* and *roX2* in *Drosophila* (Bell et al., 2018)	Bell et al., 2018
RNA and DNA interacting complexes ligated and sequenced (RADICL-seq)	To identify lncRNA-chromatin interactions	RNase H and actinomycin D decrease bias for nascent transcripts; improved genomic coverage and unique mapping efficiency	Moderate sensitivity might decrease detection of low abundance chromatin-associated RNAs	MALAT1 (Bonetti et al., 2020)	Bonetti et al., 2020
Chromosome conformation capture (3C)	Characterizing spatial topology of long-range DNA interactions	High throughput; many variations have emerged	Risk of artifacts during data analysis	ANRIL (Nakaoka et al., 2016)	Dekker et al., 2002a; Lieberman-Aiden et al., 2009; Han et al., 2018
RNA Immunoprecipitation (RIP)	Identifies lncRNA-protein interactions	Sensitive and specific for RNA detection	Need specific antibodies for protein targets	HOTTIP (Hu et al., 2019)	Lerner and Steitz, 1979; Ule et al., 2018
Cross-linking and immunoprecipitation (CLIP)	Identifies lncRNA-protein interactions	No nucleases used; do not need special reagents/equipment	UV light can cause mutations; low sensitivity	NEAT1 (Wen et al., 2020)	Ule et al., 2003
RNA pull-down	Identifies lncRNA-protein interactions	Improved discovery of weak/transient binding	Artificially increasing lncRNA of interest may generate false positives	STEEL (Man et al., 2018)	Marín-Béjar and Huarte, 2015
Chromatin immunoprecipitation (ChIP)	Identifies proteins associated with specific genomic regions	Can identify histone proteins and histones with modifications (e.g., methylation, acetylation)	Need specific antibodies	HOTTIP and ANRIL (Nakaoka et al., 2016; Hu et al., 2019)	Massie and Mills, 2012; Xie et al., 2016
RNA fluorescence *in situ* hybridization (RNA FISH)	Visualization of subcellular localization of lncRNA	Branched chain approaches can detect low abundance lncRNAs with single-cell resolution	Hybridization artifacts	MEG3 (Cabili et al., 2015)	Gall and Pardue, 1969
RNA interference (e.g., siRNA, shRNA)	Guilt by association defined by lncRNA knockdown	High knockdown efficiency	Potential off-target effects could lead to decreased specificity; transfection method artifacts	STEEL (Man et al., 2018)	Dorsett and Tuschl, 2004; Taxman et al., 2006
Antisense oligonucleotides (ASOs)	To silence lncRNA in order to assess function	Ideal for targeting non-coding nuclear RNAs	Potential off-target effects could lead to decreased specificity	MALAT1 (Gong et al., 2019)	Crooke, 2017
Clustered regularly interspersed short palindromic Repeats (CRISPR)	Ablate native lncRNA locus	Can edit any regulatory element (e.g., enhancer, promoter, etc.); no mediator machinery involved	Need to have “CRISPRable” genomic locus; challenge to study primary human cell types	MANTIS, PRANCR (Leisegang et al., 2017; Cai et al., 2020)	Jinek et al., 2012

All-to-all approaches enable global detection of RNA-chromatin interactions across all RNAs. These methods include mapping RNA-genome interactions (MARGI), global RNA interaction with DNA sequencing (GRID-seq), and chromatin-associated RNA sequencing (ChAR-seq) (Li X. et al., 2017; Sridhar et al., 2017; Bell et al., 2018). More recently, RADICL-seq (RNA and DNA interacting complexes ligated and sequenced) was developed (Bonetti et al., 2020). These techniques are based on a bivalent linker in which one end ligates to an RNA and the other end ligates to a restriction-digested DNA. Specifically, MARGI maps chromatin-RNA interactions through ligation of a lncRNA to its target genomic sequences, generating RNA-DNA chimeric sequences prior to sequencing. GRID-seq and ChAR-seq are very similar but GRID-seq employs a linker with 2 restriction sites for a type IIS restriction enzyme, MmeI, followed by digestion to produce size-specific products. On the other hand, ChAR-seq employs sonication of ligated products to generate smaller fragments prior to library preparation. RADICL-seq is similar to GRID-seq in that its linker also has 2 restriction sites (for *Eco*P15I), but it also uniquely employs RNase H and actinomycin D to reduce bias toward abundant nascent transcripts. These all-to-all methods are limited by their moderate sensitivity, which could decrease the detection of low abundance chromatin-associated RNAs.

To visualize 3D chromatin interactions, chromosome conformation capture (3C) has historically been used to characterize long-range DNA contacts (Dekker et al., 2002a; Han et al., 2018). Many adaptations of this technique exist, including chromosome conformation capture-on-chip (4C), or chromosome conformation capture carbon copy (5C). These variations require knowledge of the target loci, but another adaptation referred to as Hi-C, provides unbiased locations of chromatin interactions across the genome (Lieberman-Aiden et al., 2009).

To study lncRNA-protein interactions, scientists utilize RNA immunoprecipitation (RIP), cross-linking and immunoprecipitation (CLIP), and RNA pull-down (Lerner and Steitz, 1979; Ule et al., 2003, 2018; Marín-Béjar and Huarte, 2015). Another immunoprecipitation-based approach is chromatin immunoprecipitation (ChIP) which identifies protein-DNA interactions (Massie and Mills, 2012; Xie et al., 2016). These techniques have further advanced in recent years. For example, RIP can be combined with APEX (engineered ascorbate peroxidase)-catalyzed proximity biotinylation of endogenous proteins (APEX-RIP) to improve the spatial resolution of RNA mapping (Kaewsapsak et al., 2017). A recent study used APEX-RIP to generate a transcriptome-wide RNA atlas (Fazal et al., 2019).

Another powerful technique is RNA fluorescence *in situ* hybridization (FISH) which allows researchers to visualize RNA and DNA molecules while retaining cell morphology (Gall and Pardue, 1969; Collins et al., 1997; Cabili et al., 2015). The quantitative strength of this assay has increased with improvements of the branched DNA signal amplification technology to amplify the signal 1,000–10,000-fold. FISH improves the detection of lowly expressed lncRNAs. Recently, fluorescence in situ RNA sequencing (FISSEQ) was developed (Lee et al., 2015). FISSEQ provides high throughput information on tissue-specific gene expression while maintaining spatial context.

To silence lncRNAs and study their functions, there are many well-established techniques. There is RNA interference (RNAi), which is widely used, and is very efficient at knocking down RNAs (Watts and Corey, 2012; Chery, 2016). RNAi works best at targeting cytoplasmic lncRNAs. To silence nuclear lncRNAs, antisense oligonucleotides (ASOs) can be used in which RNase H is recruited to hydrolyze RNA in a DNA: RNA complex, causing transcriptional silencing of the target lncRNA. Finally, there is clustered regularly interspaced short palindromic repeats (CRISPR) which is similar to RNAi except that it is not reliant on mediator machinery (Jinek et al., 2012; Awwad, 2019). CRISPR can directly target genomic regions, allowing scientists to target regulatory regions like promoters or enhancers. CRISPR interference (CRISPRi) has been used for large-scale, systematic lncRNA screens in cell lines, demonstrating how this tool can be used to identify lncRNAs and further study their functions (Koch, 2017; Liu et al., 2017, 2020; Cai et al., 2020). Importantly, not all lncRNAs can be studied using CRISPR because CRISPR efficiency is affected by internal or bidirectional promoters (Goyal et al., 2017).

LncRNAs are a highly heterogeneous and functionally diverse class of molecules. As we illustrate in this section, there is a growing number of methods that enable functional characterization of lncRNAs. Many of these methods can be combined to efficiently map the lncRNA interactome. The data generated are increasingly being archived on a multitude of public databases. We direct readers to reviews that outline existing lncRNA databases (Peng et al., 2020; Pinkney et al., 2020). There is no doubt that high-throughput technologies will continue to advance to produce higher quality lncRNA data and improve our overall understanding of their molecular functions.

## Angiogenesis

The *de novo* formation of blood vessels from angioblasts and circulating hematopoietic stem cells is called vasculogenesis (Cines et al., 1998). In the absence of healthy vascular development, embryonic lethality results. Post-natally, new blood vessels form from pre-existing blood vessels in a closed cardiovascular system in a process called angiogenesis. Angiogenesis plays an important role in development, wound healing, and many other physiological processes (Schmidt and Carmeliet, 2010). ECs are instrumental in the orchestration of angiogenesis. It is a highly choreographed cascade of events that involves both exogeneous (e.g., hypoxia and VEGF) and endogenous regulatory inputs. There are two main types of angiogenesis: sprouting angiogenesis and intussusceptive angiogenesis. In sprouting angiogenesis, as the name suggests, new blood vessels grow via “sprout” formation from existing vessels through EC proliferation and migration (Ackermann et al., 2014). In contrast, intussusceptive angiogenesis, also referred to as splitting angiogenesis, there is little dependence on EC proliferation and migration. Instead, the ECs reorganize, and the cells invade the lumen forcing the vessel to split (Konerding et al., 2012). Both forms of angiogenesis, which are believed to occur in nearly all organs and tissues, produce new vasculature, most often in capillary beds (Adair and Montani, 2010). Sprouting angiogenesis, hereafter angiogenesis, is better understood and the most well-studied. As such, it will be the focus of this review.

**FIGURE 2:**
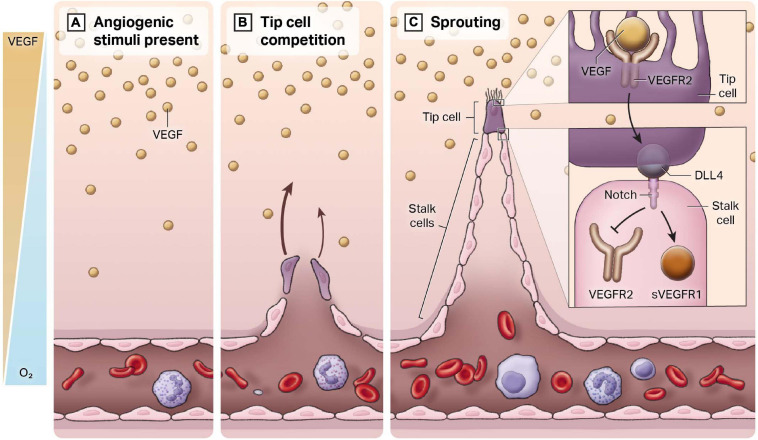
Endothelial heterogeneity in the angiogenic response. New blood vessels develop from a pre-existing closed cardiovascular system, a process termed angiogenesis. **(A)** In regions of hypoxia, there is an increase in the release of the pro-angiogenic ligand VEGF. An inverse gradient is established between free oxygen concentration and VEGF. **(B)** Quiescent endothelial cells respond to VEGF and compete to be the leader or the “tip” of the newly forming blood vessel. **(C)** The cell that becomes the tip cell represses expression of tip markers in adjacent cells, which are known as stalk cells. Stalk cells proliferate to form the body of the vessel.

A multitude of factors work together in a dynamic network to maintain tight regulation of angiogenesis. This is to prevent insufficient or over-vascularization from occurring. A major environmental regulator is hypoxia, which can be defined as an imbalance between oxygen supply and demand. The main intracellular signaling molecule is hypoxia inducible factor (HIF), a transcription factor comprised of HIF-alpha and HIF-beta subunits (Shweiki et al., 1992). The HIF-alpha subunit is functionally regulated by oxygen-dependent post-translational modifications of prolyl residues. In normoxia, the prolyls are hydroxylated, and Von Hippel-Lindau (VHL) recruits the E3 ubiquitin ligase complex to ubiquitinate the HIF-alpha subunits and make them a target for proteasomal degradation (Chen et al., 2009). In contrast, in hypoxic conditions, there is no hydroxylation and therefore no subsequent ubiquitination. Thus, HIF is not degraded and instead accumulates and translocates to the nucleus. Together with the constitutively expressed HIF-beta, it can bind to the hypoxia-responsive cis-DNA element (HRE) and modify transcription of a number of genes, many of which are involved in angiogenesis. This includes genes like matrix metalloproteinase-2 (MMP-2), angiopoietin-2 (Ang-2), Tie-2, PDGF, Delta-like 4 (DLL4) and many more factors. However, the most important target is arguably vascular endothelial growth factor (VEGF). The development of normal vasculature is heavily dependent on a VEGF gradient (see Figure 2). When the gene dose of VEGF is reduced by 50%, it causes embryonic lethality due to vascular deficiencies (Carmeliet et al., 1996; Ferrara et al., 1996). Conversely, overexpression of VEGF, as seen in tumors, causes exuberant EC activation, leading to a disorganized vasculature (Jain, 2005). Since VEGF is a potent regulator of angiogenesis, VEGF is often targeted therapeutically to treat diseases in which this process is dysregulated.

In healthy adult blood vessels, vascular ECs exhibit a low rate of cell number turnover (Sender and Milo, 2021). Adult humans exhibit approximately 1 trillion ECs, with a mean life span typically of 3–10 years. This long-life span can be contrasted with red blood cells or circulating white blood cells, which approximate 120 and 7 days, respectively. Importantly, the basal low rate of turnover of ECs can be markedly augmented. Our current understanding of angiogenesis can be encapsulated by the tip-stalk paradigm (see Figure 2). Once hypoxia has helped establish the VEGF gradient from non-endothelial cell types (e.g., macrophages), ECs respond and begin to migrate toward this angiogenic stimulus. These ECs are called “tip cells,” and they are morphologically characterized by filopodia and stress fibers, which facilitate invasion into the surrounding tissue and creates a clear path for sprouting to commence (Eilken and Adams, 2010). VEGF binds to vascular endothelial growth factor receptor 2 (VEGFR2) receptors in tip cells, activating DLL4, which then binds to Notch receptors in adjacent ECs (Adams and Alitalo, 2007). Notch signaling is activated in adjacent ECs and suppresses tip genes, such as VEGFR2 and DLL4, to prevent these ECs from also becoming tip cells. These cells are referred to as “stalk cells.” In addition to positional identity, stalk cells are highly proliferative, lack filopodia and contribute to lumen formation. They express soluble VEGFR1 (sVEGFR1), which sequesters VEGF-A in a regulatory manner to prevent VEGF-induced signaling (del Toro et al., 2010).

Once multiple sprouts have formed, tip cells of different sprouts will anastomose with each other, which is believed to occur through filopodial interactions (Bentley et al., 2009). This creates vessel networks. Wnt signaling is also important in angiogenesis. Specifically, the interplay between Notch signaling and Wnt signaling causes an upregulation of β-catenin expression. β-catenin is important in stabilizing tight junctions and activating PDGF-B expression. PDGF-B promotes recruitment of mural cells/support cells, which is an indicator of healthy and mature vessels and prevents vessel “leakiness” (Gavard and Gutkind, 2008; Reis et al., 2012). Lumenization and blood flow will further stabilize these new vessel connections (Chappell et al., 2011; Potente et al., 2011). Once the vessels have been perfused, the ECs will move toward a quiescent phenotype. It is important to note that the underlying mechanisms, in particular of these final phases of sprouting angiogenesis, are not well understood.

As inferred from above, hemodynamic forces must regulate angiogenesis. In regions of the body in which angiogenesis occurs such as in muscle that undergoes remodeling following exercise, there are markedly increased levels of shear stress. In contrast, there is low shear stress in a tumor. Of note, angiogenesis occurs at both these levels of shear stress, though in opposite directions (Kaunas et al., 2011). In normal physiological conditions, shear stress varies depending on the vessel type. In arteries, shear stress can range between 10 and 70 dynes/cm^2^, whereas in veins, shear stress can range between 1 and 6 dynes/cm^2^ (Lipowsky, 1995; Malek, 1999). Thus, it is not the absolute value of shear stress that induces angiogenesis, but instead, the deviation from normal levels detected by the ECs that stimulates this physiological response.

## Nuclear Endothelial-Enriched Angiogenic lncRNAs

In the last several years, lncRNAs that regulate biological processes like angiogenesis have emerged. In this section, we will highlight angiogenic lncRNAs that act through chromatin-based mechanisms to effect angiogenesis, including STEEL, GATA6-AS, and MANTIS (Table 3). These lncRNAs are also of particular interest because they are regulated by environmental stimuli that regulate angiogenesis: hemodynamic forces and hypoxia.

**TABLE 3:** Summary of endothelial-enriched angiogenic lncRNA function.

LncRNA	Epigenetic mechanism of action	Function in angiogenesis	References
STEEL	In static conditions, STEEL is recruited to EC proximal promoter regions, eNOS and KLF2, where STEEL’s association will enhance transcription.	STEEL increases angiogenesis *in vitro* and *in vivo.*	Man et al., 2018
GATA6-AS	Hypoxia-responsive GATA6-AS interacts with LOXL2 and deactivates H3K4me3 to repress transcription of COX-2 and POSTN.	GATA6-AS increases sprouting *in vitro* but decreases blood vessel formation *in vivo.*	Neumann et al., 2018
MANTIS	When JARID1B is repressed, MANTIS increases and associates with BRG1, which is associated with the SWI/SNF remodeling complex and is stabilized by BAF155.	MANTIS increases transcription of pro-angiogenic factors (i.e., COUP-TFII, SMAD6, SOX18) *in vitro*.	Leisegang et al., 2017, 2019

### STEEL

STEEL was the first flow-regulated, endothelial-enriched lncRNA identified. STEEL is a pro-angiogenic lncRNA that links decreased laminar shear stress with pro-angiogenic programming. It is downregulated by laminar flow (10 dynes/cm^2^) and enriched in microvascular ECs (Man et al., 2018). In static conditions, STEEL maintains eNOS and KLF2 expression at basal levels required for angiogenesis. Consistent with its nuclear localization, we found evidence that STEEL regulates eNOS and KLF2 by transcriptional mechanisms. First, STEEL knockdown decreased heterogeneous nuclear RNA (hnRNA) levels and decreased RNA polymerase II (Pol II) loading at the proximal promoters of eNOS and KLF2. Moreover, STEEL regulates chromatin accessibility, nucleosome occupancy and histone 3 lysine 4 trimethylation (H3K4me3) at both eNOS and KLF2 proximal promoters. Interestingly, a feedback loop exists in which laminar shear stress (10 dynes/cm^2^) induces high levels of eNOS and KLF2, which in turn represses STEEL expression. In this way, STEEL functions as a rheostat of angiogenesis that responds to shear stress conditions. Mechanistically, they identified a lncRNA-protein interaction that presents a new mechanism for genomic targeting of the poly-ADP ribosylase 1 (PARP1), which contributes to transcriptional regulation, DNA damage repair, and cardiovascular disease (Chaudhuri and Nussenzweig, 2017). Using RNA pulldown followed by mass spectrometry and RIP to identify and confirm this interaction, respectively, and ChIP to demonstrate an effect of STEEL knockdown on PARP1 occupancy at the eNOS and KLF2 promoters. Together, these mechanistic studies of the STEEL lncRNA provide evidence for epigenetic regulation of gene expression and novel lncRNA-protein interactions. The underlying mechanisms of these interactions require further investigation.

Regarding its angiogenic functions, STEEL was shown to affect blood vessel formation both *in vitro* and *in vivo.* Using a Matrigel network assay in HUVEC, STEEL knockdown decreased network formation while STEEL overexpression increased network formation (Man et al., 2018). Cell migration is characteristic of tip cells and cell proliferation is characteristic of stalk cells. Both assessments are key measures of sprouting angiogenesis and were therefore examined. As expected, through scratch wound assay, carboxyfluorescein succinimidyl ester (CFSE) labeling and bromodeoxyuridine (BrdU) incorporation, STEEL overexpression promoted EC proliferation and migration. These results were confirmed *in vivo* using a mouse model. Collagen modules with stromal cells were coated with either control or STEEL overexpressing ECs transduced and implanted into immunocompromised mice. Using micro-computed tomography (micro-CT) imaging, it was found that STEEL-transduced implants had more vessels that were perfused compared to control implants, which not only had fewer vessels, but also demonstrated extravasation and pooling. Further examination of the STEEL-transduced implants’ vascular networks revealed that the blood vessels showed mural cell support, as indicated by smooth muscle actin staining, demonstrating vessel maturity. Of note, STEEL-induced angiogenesis did not display evidence of morphologically abnormal vessels (e.g., contrast leakage, lack of pericyte coverage).

### GATA6-AS

Another endothelial-enriched lncRNA is the antisense transcript of GATA6 (GATA6-AS). GATA6-AS is upregulated approximately 2.5-fold under chronic hypoxia (24 h) and localized primarily to the nucleus (Neumann et al., 2018). Using mass spectrometry, it was found that GATA6-AS interacts with lysyl oxidase-like 2 (LOXL2), a known hypoxia regulator (Bignon et al., 2011). Nuclear LOXL2 is a known co-repressor of transcriptional activity and deactivates H3K4me3. With GATA6-AS repression, there was a 30% decrease in H3K4me3, which also occurred under hypoxia. Curiously, when LOXL2 was repressed, H3K4me3 was increased, and the majority of GATA6-AS regulated genes were inversely expressed when compared to GATA6-AS repression. Cyclooxygenase-2 (COX-2) and periostin (POSTN) were more closely examined with ChIP-PCR. COX-2 catalyzes the production of prostaglandins in ECs, which contributes to flow-mediated vasodilation whereas POSTN acts through Erk/VEGF signaling to stimulate angiogenesis (Koller et al., 1993; Duffy et al., 1998). GATA6-AS silencing markedly decreased H3K4me3 at the promoter regions of both these genes, pointing toward an epigenetic role for GATA6-AS on EC gene expression.

It was argued that GATA6-AS regulates angiogenesis through endothelial-to-mesenchymal transition (EndMT), a process that can be induced by hypoxia (Neumann et al., 2018). Using an EndMT-assay, repressing GATA6-AS in HUVECs largely inhibited EndMT. Further *in vitro* analysis of angiogenesis using a spheroid assay showed that GATA6-AS silencing significantly decreased sprouting. GATA6-AS repression decreased cell migration, but it did not affect proliferation or apoptosis. The effect GATA6-AS has on the EndMT process may in turn, be affecting angiogenic potential. Using an *in vivo* immune deficient mouse model, HUVEC transfected with control GapmeRs or GapmeRs against GATA6-AS were transplanted. Through histological visualization, GATA6-AS repressed cells had a marked increase in the number of perfused, mature blood vessels compared to controls. These *in vivo* findings are contradictory to the *in vitro* results. The decrease in sprouting *in vitro* may be compensated for through other mechanisms *in vivo.* LOXL2 in the extracellular matrix is also involved in angiogenesis. When LOXL2 was repressed, there was a decrease in sprouting. Interestingly, LOXL2 did not decrease with GATA6-AS silencing. It is clear GATA6-AS is regulating angiogenesis, but the mechanisms require further study.

### MANTIS

MANTIS is a flow-regulated lncRNA expressed by ECs. This nuclear lncRNA was identified through inhibition of an EC-enriched H3K4 lysine-specific demethylase 5B (JARID1B) (Leisegang et al., 2017). MANTIS is not specific to ECs; it is also expressed by smooth muscle cells. Steady, laminar flow upregulates MANTIS and is mediated through KLF2 and KLF4 (Leisegang et al., 2019). Moreover, when MANTIS was repressed, HUVEC were unable to align in the direction of flow. Using ChIP, JARID1B was bound to an H3K4me3 region near the MANTIS transcription start site (TSS), which was further increased with JARID1B silencing. Using mass spectrometry, MANTIS was found to be highly associated with Brahma Related Gene 1 (BRG1), an ATPase involved in the SWItch/Sucrose Non-Fermentable (SWI/SNF) chromatin remodeling complex and important for EC function. BRG1 is stabilized by BAF155. With MANTIS silencing, there was marked reduction in BRG1 and BAF155 binding and the ability of BRG1 to bind to target promoters.

Using CRISPR/Cas9, MANTIS was functionally inactivated in HUVEC, resulting in significantly less tube formation and sprouting. Silencing MANTIS yielded similar results and also decreased cell migration. This also resulted in decreased mRNA and protein expression of factors important in angiogenesis including chicken ovalbumin upstream promoter – transcription factor 2 (COUP-TFII), SMAD6 and sex determining region Y-box 18 (SOX18). It should be noted that there are many other tip and stalk genes that are also relevant that were not assessed in this study. When these 3 factors are reduced, there is decreased sprouting and yet, overexpression of these factors does not restore sprouting to normal. However, the regulation of these proteins through MANTIS may be important in maintaining healthy sprouting in ECs. MANTIS was knocked down and ATAC-Seq was conducted. BRG1 protein levels were unchanged, but at the TSS of COUP-TFII, SMAD6 and SOX18, there was a decrease in open chromatin. Using micrococcal nuclease (MN) digestion, there was an increase in nucleosomal formation at the TSS of these 3 genes when MANTIS was decreased. MANTIS repression increased H3K27me3, and decreased RNA Pol II at the TSS of all 3 genes. In addition, silencing MANTIS reduced BRG1 binding at the TSS of COUP-TFII, SMAD6 and SOX18. Since BRG1 is known to play a role in nucleosome remodeling, this may suggest that BRG1’s interactions with these proteins may be mediated through MANTIS. Further study of MANTIS is needed to better understand these interactions.

## Angiogenic lncRNAs in Disease

The majority of lncRNAs that have been identified and characterized have been in diseases. This include cancers (Jin et al., 2020; Teppan et al., 2020; Zhou et al., 2020; Katsushima et al., 2021), diabetes (Taheri et al., 2020; Xu E. et al., 2020; Ismail et al., 2021), cardiovascular diseases (Fang et al., 2020; Meng et al., 2020; Yeh et al., 2020) and ischemic stroke (Gan et al., 2021; Wolska et al., 2021). In this section, we will focus on angiogenic lncRNAs enriched in disease and their epigenetic functions. We will highlight some of the most extensively studied lncRNAs: MALAT1, MEG3 and ANRIL. A summary of these as well as other more recently published disease-associated angiogenic lncRNAs can be seen in Table 4 (Zhou et al., 2016; Li Y. et al., 2017; Ruan et al., 2018; Niu et al., 2020; Xu X. et al., 2020; Zhang H. et al., 2020; Biswas et al., 2021).

**TABLE 4:** Summary of disease-associated angiogenic lncRNA function.

LncRNA	Disease	Epigenetic mechanism of action	Function in angiogenesis	References
HOX transcript antisense RNA (HOTAIR)	Diabetic retinopathy	Histone methylation, histone acetylation, DNA methylation	HOTAIR regulates glucose-mediated increases of angiogenesis in diabetic retinopathy	Biswas et al., 2021
Small nucleolar RNA host gene 14 (SNHG14)	Hepatocellular carcinoma	SNHG14 upregulates PABPC1 expression via H3K27 acetylation	SNHG14 promotes proliferation and tube formation in endothelial cells	Zhang H. et al., 2020
LINC00337	Colorectal cancer	LINC00337 recruits DNMT1 to CNN1 promoter, which inhibits its transcription and increases VEGF-mediated angiogenesis	LINC00337 increases tumor growth and microvascular density	Xu X. et al., 2020
RAB11B Antisense RNA 1 (RAB11B-AS1)	Breast cancer, osteosarcoma	RAB11B-AS1 increases RNA Pol II in hypoxia to upregulates VEGFA and ANGPLT4	HIF2 induces RAB11B-AS1 which increases angiogenic factors	Niu et al., 2020
Metastasis associated lung adenocarcinoma transcript 1 (MALAT1)	Multiple cancers	Formation of molecular scaffolds, splicing and regulating histones and transcription factors	MALAT1 increased proliferation, sprouting and migration in ECs	Li et al., 2017
Maternally expressed 3 (MEG3)	Idiopathic pulmonary fibrosis, cholestatic liver injury	MEG3 interacts with JARID2 which recruits PRC2	MEG3 regulates NOTCH and VEGF pathways	Ruan et al., 2018
Antisense non-coding RNA in the INK4 locus (ANRIL, CDKN2B, CDKN2B-AS1)	Coronary heart disease, ischemic stroke, type 2 diabetes, atherosclerosis	Promoter methylation, chromatin modifications, alternative splicing and post-transcriptional modifications	High glucose upregulates ANRIL in retinal ECs and is involved in VEGF regulation	Zhou et al., 2016

### MALAT1

MALAT1, as the name suggests, is implicated in protean cancer cell types. It is extremely abundant in multiple cell types, including vascular ECs (Gutschner et al., 2013; Michalik et al., 2014; Yan et al., 2016). Primarily localized in the nucleus as part of nuclear speckles, MALAT1 associates with the serine/arginine (SR) family of pre-mRNA splicing factors such as SRSF1/2/3; it plays an important role in alternative splicing. When MALAT1 is silenced, it results in reduced nuclear speckle association of many pre-mRNA splicing factors including SF1, U2AF65, SF3a60, and U2snRNP *in vitro* (Tripathi et al., 2010). MALAT1 may have species-specific function. Unexpectedly, Malat1 knockout mice evidenced no change in nuclear speckle markers compared to wildtype mice (Nakagawa et al., 2012). This finding in mice was confirmed by other studies (Eißmann et al., 2012; Zhang et al., 2012).

MALAT1 also plays a critical role in transcriptional regulation, through direct binding to the 3′ end of actively transcribing gene bodies, and mediating localization of unmethylated proteins in nuclear speckles (Engreitz et al., 2014). MALAT1 functions as a molecular scaffold for unmethylated polycomb 2 proteins (PC2), E2F transcription factor, and histones involved in active transcription and the transcriptional coactivator complex (Yang et al., 2011). MALAT1 has a role in regulating expression of cyclins and cell cycle kinases. Specifically, it regulates S-phase cyclins, p21 and p27Kip1 in mouse (Michalik et al., 2014). Overall, MALAT1’s abundance in the cell, varied half-life and structural stability conferred by its 3′ end triple-helix structure, contributes to its functional stability and diversity.

In gastric cancer, MALAT1 promotes vascular mimicry and angiogenesis to establish tumorigenicity and metastasis (Li Y. et al., 2017). When MALAT1 is repressed in HUVEC, ECs were no longer able to form vessels via the tube formation assay. Knockdown was also able to increase EC permeability. With hypoxia, MALAT1 is upregulated and enhances proliferation of ECs *in vitro*. In another study by Michalik et al., MALAT1 knockdown in ECs increased sprouting and migration, but decreased stalk cell proliferation via cell cycle inhibition. Examining a mouse knockout of Malat1, scientists found no affect in adults, but it reduced vascular proliferation and network formation in embryonic retina. In the hind limb ischemia model, Malat1 deficiency decreased neovascularization, capillary density and recovery of blood flow. In thyroid tumors, MALAT1 promotes Fibroblast Growth Factor 2 (FGF2) secretion from tumor-associated macrophages into the tumor microenvironment to mediate angiogenesis (Huang et al., 2017). Together, the role of MALAT1 in angiogenesis is conferred by its role in alternative splicing, molecular scaffold formation and binding to actively transcribed gene loci, in particular the cell cycle genes. Clearly, MALAT1’s functions are diverse.

### MEG3

Maternally expressed gene 3 (MEG3) is a nuclear and EC-enriched lncRNA that exhibits multiple mRNA transcript variants (Zhang et al., 2010). It is also an imprinted gene (Michalik et al., 2014). Imprinting is an epigenetic phenomenon in which monoallelic silencing of some genes occurs in a parent-of-origin specific manner (Autuoro et al., 2014). This process is thought to be regulated by lncRNAs, though the mechanisms have yet to be fully elucidated. MEG3 is encoded by the imprinted *DLK1-DIO3* locus, and it was found that it interacts with Jumonji And AT-Rich Interaction Domain Containing 2 (JARID2), an important component of PRC2 in pluripotent stem cells (Kaneko et al., 2014). This interaction is needed in order to recruit and assemble PRC2 at a subset of pluripotent stem cell genes. This suggests that the interplay of these RNA-based interactions may participate in the epigenetic regulation of genes involved in the process of transitioning stem cell pluripotency to differentiation. MEG3’s binding sites also have GA rich regions critical to guiding MEG3 to chromatin through the formation of RNA-DNA triplex structures (Mondal et al., 2015).

MEG3 was shown to be among the top 10 most abundant lncRNAs in HUVEC, strongly suggesting a clear biological role in ECs (Michalik et al., 2014). It also inhibits VEGF and Notch pathways, which we know are important signaling pathways in angiogenesis (Gordon et al., 2010). Adding to this, MEG3 expression is also upregulated by hypoxia. Ruan et al. overexpressed constitutive HIF-1alpha and found increased activity in the MEG3 promoter. Next, they examined chronic treatment (24 h) of pro-angiogenic growth factors like VEGF, bFGF and Transforming Growth Factor β (TGFβ). There was no effect on MEG3 expression, but there were still notable angiogenic effects. MEG3 knockdown markedly decreased VEGFR2 mRNA and protein expression in HUVEC, which then inhibited cell migration. Moreover, it impaired the ability of ECs to form tube-like structures and significantly decreased sprouting from spheroids in both normoxic and hypoxic conditions (Ruan et al., 2018). It also was found that genes of the TGFβ signaling pathway are direct targets of MEG3 and that MEG3 binds to distal regulatory sites of these genes. Thus, even though there was not a direct effect with pro-angiogenic factors, the downstream factors of these angiogenic pathways are still indirectly regulated by MEG3.

MEG3 can be characterized as a tumor suppressor important in cell cycle regulation and apoptosis (Li et al., 2015). Long-range interaction between distal loops of MEG3 secondary structure forms a pseudoknot which allows MEG3 to upregulate p53 expression (Li et al., 2015; Uroda et al., 2019). MEG3 allelic loss of locus is associated with meningioma pathogenesis and progression (Zhang et al., 2010). Expression of MEG3 in human meningioma cell lines clearly shows marked suppression of tumor cell growth and activation of p53. MEG3 also regulates age-associated decline in endothelial function; MEG3 was significantly upregulated in senescent HUVEC (passages 16–18) compared to earlier HUVEC passages (3–4) (Boon et al., 2016; He et al., 2017; Wu et al., 2017). Scientists found that when Meg3 was repressed in HUVEC, age-mediated inhibition of sprouting was stopped, implying that Meg3 silencing could be a potential way to rescue age-associated impairments in angiogenic potential. In the brain, Meg3 null mice exhibit enhanced vascular density (Gordon et al., 2010). Examining this closer, Meg3 null mice, showed increased VEGFA, VEGFR1, DLL4, among other angiogenic genes. It was previously shown that p53 could bind to Sp1 sites in the VEGFA promoter to negatively regulate VEGFA transcription (Pal et al., 2001). Thus, loss of MEG3 may decrease p53 binding, thereby causing an increase in transcription of genes involved in VEGF signaling. Clearly, MEG3 has an important role as an angiogenic regulator.

### ANRIL

ANRIL (also known as CDKN2B or CDKN2B-AS1) is located on chromosome 9p21. GWAS identified this disease-associated locus as a “protein gene desert” (Cheng et al., 2005; Iaconetti et al., 2013; Wahlestedt, 2013; Carninci et al., 2021). ANRIL is transcribed antisense to the INK4b-ARF-INK4a gene cluster (Derrien et al., 2012). Exons 13-19 of ANRIL overlapped with a high-risk haplotype associated with genetic predisposition to coronary artery disease (CAD) (Broadbent et al., 2008). A genetic association is also evident with ischemic stroke, aneurysms, and peripheral vascular diseases (Zeggini et al., 2007; Foroud et al., 2012; Kremer et al., 2015; Kong et al., 2016; Tan et al., 2019). ANRIL is especially enriched in vascular smooth muscle cells (VSMC) and mononuclear phagocytes within atherosclerotic plaques (The Encode Project Consortium, 2012; Zollbrecht et al., 2013; Bai et al., 2014; Nanda et al., 2016). ANRIL is a better genetic predictor of cardiovascular diseases than classical clinical measures such as blood pressure and dyslipidemia (Holdt et al., 2010).

ANRIL has at least 20 linear or circular isoforms associated with atherosclerosis (Burd et al., 2010; Hubberten et al., 2019). Though the mechanism(s) by which minor frequency alleles of ANRIL still predispose to disease remain to be fully elucidated, it is argued that ANRIL regulates this genomic region *in cis* whereby the risk allele leads to an increase in linear ANRIL, but reduced levels of circular ANRIL (Holdt and Teupser, 2018). Linear ANRIL may function as a scaffold for epigenetic protein complexes that stimulate pro-atherosclerotic cellular functions. ANRIL is highly enriched in the nucleus, playing an active role in chromatin modification (Zhou et al., 2016). It is regulated by promoter methylation, transcription factors, alternative splicing and post-transcriptional modifications. ANRIL interacts with PRC1 & PRC2 to epigenetically repress neighboring genes such as CDKN2A and CDKN2B *in cis*. ANRIL and CDKN2A form a scaffold with H3K27me3 with polycomb Chromobox 7″ (CBX7); ANRIL with CDKN2B interact with PRC2 subunit SUZ12 (Yap et al., 2010). *Trans* activity of ANRIL through PRC1/2 represses distant genes that are dependent on the Alu elements found in ANRIL and in target gene promoters. ANRIL creates a scaffold for WD repeat-containing protein 5 (WDR5), a histone H3K4 presenter and histone deacetylase 3 (HDAC3) coordinating histone modification on target genes of vascular smooth muscle cell phenotypes (Zhang C. et al., 2020).

ANRIL has been shown to be upregulated in human retinal ECs stimulated by high glucose and diabetes. In diabetic retinopathy, ANRIL regulates VEGF through interactions with PRC2 components p300, miR200b, and enhancer of zeste homolog 2 (EZH2) (Thomas et al., 2017). Since VEGF is involved in stimulating vascular permeability, migration and proliferation of ECs, ANRIL upregulating VEGF contributes to promoting endothelial injury, which occurs via tumor necrosis factor-alpha (TNFα)- nuclear factor kappa light-chain-enhancer of activated B cells (NFkB)-ANRIL/YY-IL6 signaling pathways (Zhou et al., 2016). Similarly, in a rat model with diabetes and cerebral infarction, overexpression of ANRIL increased VEGF expression, resulting in increased angiogenesis via NFkB signaling (Zhang et al., 2017). ANRIL also regulates Akt phosphorylation in ECs and scientists recently showed that in mice, ANRIL improves cardiac function and post-ischemic angiogenesis following myocardial infarction by upregulating angiogenesis through Akt activation (Huang et al., 2020). Finally, Zeng et al. recently showed that ANRIL levels were elevated in the serum of thrombosis patients relative to healthy patients (Zeng et al., 2019). To assess the effect of ANRIL on angiogenesis, they took Sprague Dawley rats and injected si-ANRIL and examined lumen formation. They found that there were fewer lumens and smaller lumens in the rats with repressed ANRIL relative to the control group, confirming a role for ANRIL in angiogenesis. These disease associations position ANRIL as a key target for treatment of cardiovascular disease.

## Challenges Associated With Studying lncRNAs

There are many challenges associated with studying lncRNAs. The first question when studying a lncRNA is verifying whether it is truly a bona fide lncRNA. RNA-seq data is mapped to the most recent build of the human genome. These reference databases are not comprehensive with respect to lncRNA annotation, thus limiting discovery. Moreover, lncRNAs are typically rare transcripts. Many are only expressed in specific contexts (e.g., development, disease, specific environmental stimuli, etc.), which can make discovery difficult. This review does not address the emerging concepts on the role of lncRNAs as post-transcriptional modifiers of gene expression and function. We and others have recently addressed the cytoplasmic function of lncRNAs (Rashid et al., 2016; Noh et al., 2018; Aillaud and Schulte, 2020; Ho et al., 2021). We also acknowledge that a key concept, and one that warrants deeper study, is the shuttling of lncRNAs in and out of the nucleus. Moreover, the varied RNA transcripts derived from specific lncRNA genes may have distinct subcellular locations. It is paramount that the candidate lncRNA structure and diversity be assessed before proceeding with detailed mechanistic and functional studies.

The paradigm that a gene must have either protein-coding or a non-coding function, but not both, is too simplistic. Some RNAs have both coding and non-coding functions (Robb et al., 2004; Fish et al., 2007). The sONE RNA, is a lncRNA antisense to eNOS that exhibits exon/exon sense/antisense interactions. The sONE locus also has a minor mRNA variant that encodes a protein involved in the autophagy pathway. Human ECs have high levels of eNOS mRNA, but low levels of sONE RNA. When sONE RNA is overexpressed, there is decreased eNOS mRNA and protein expression. Adding to this, sONE RNA is upregulated by hypoxia in ECs and VSMCs. Notably, sONE is primarily localized to the nucleus in normoxia, but with hypoxia, the sONE RNA is shuttled into the cytoplasm.

LncRNAs exhibit protean intra-species allelic diversity. Furthermore, it follows and has been noted that lncRNAs have low inter-species sequence conservation, likely due to rapid evolutionary turnover (Hezroni et al., 2015; Quinn et al., 2016). For lncRNA homologs, generally the length of an alignable sequence is about 5 times shorter than that of a protein-coding gene. A normal lncRNA that is conserved between humans and mice will have about 20% interspecies homology, which decreases to about 5% in fish. As a result, lncRNAs may be absent in model organisms, making it hard for scientists to not only discover lncRNAs, but also to assess their *in vivo* function. There is some sequence conservation in lncRNAs, typically in short sequence islands, and perhaps this is because these are regions that are required for specific interactions with other RNAs, proteins or DNA (Kapusta and Feschotte, 2014; Quinn et al., 2016; Ulitsky, 2016). However, there are other factors to consider in the discussion of conservation besides sequence similarity alone. In fact, scientists have identified many orthologous RNAs with highly divergent sequences, that they would no longer be identifiable as orthologs by sequence similarity alone, but their function is preserved (Ponjavic et al., 2007; Ulitsky et al., 2011; Ulitsky, 2016). Another factor is positional conservation in which lncRNAs can be detected from syntenic loci even in the absence of most, if not all sequence similarity. It is clear that we need to define lncRNA conservation traits/signals. Another debated factor is structural conservation. Scientists have shown that there is limited association between secondary structure and sequence conservation (Managadze et al., 2011; Yang and Zhang, 2015). Moreover, there is evidence that specific lncRNAs act through specific tertiary or quaternary structural features, such as the triplex elements at the 3′-termini of MALAT1 or Nuclear Enriched Abundant Transcript 1 (NEAT1) (Wilusz et al., 2012). Further study on lncRNA structural conservation is evidently needed to improve our understanding on inter- and intra-species lncRNA conservation.

## New Frontiers: lncRNAs as Diagnostic and Therapeutic Targets in Medicine

As we look toward the next decade of lncRNA research, it will be interesting to see more clinical studies evaluating whether lncRNAs have the potential to be used as biomarkers or therapeutic targets for clinical interventions to improve disease outcomes. LncRNAs are lowly expressed, so we know quantification in biological fluids will be challenging. Moreover, they are poorly conserved across species making them difficult to study using *in vivo* models of disease. However, lncRNAs can be highly tissue-specific, which sets up these molecules to be very specific biomarkers. To date, prostate cancer antigen 3 (PCA3) is the only lncRNA approved as a clinical diagnostic biomarker for early detection of prostate cancer (Groskopf et al., 2006).

As for targeting lncRNAs, many scientists agree that the key will be through identifying the optimal delivery system. There has been growing interest in recent years in extracellular vesicles. Though we did not discuss it in this review, many lncRNAs, especially those expressed in cancer, have also been shown to be secreted by extracellular vesicles (Wu et al., 2017; Tellez-Gabriel and Heymann, 2019; Zhao et al., 2019; Fan et al., 2020). Extracellular vesicles are of interest because they are encapsulated by a lipid bilayer, which overcomes concerns with stability. In addition, extracellular vesicles have less immunogenicity and higher *in vivo* stability compared to widely used viral and non-viral vectors (Chen et al., 2021). Exosomes, a subtype of extracellular vesicles, are currently being examined. They have poor efficiency with respect to packaging large nucleic acids, but this is overcome through integration with liposomes or nanoparticles, which improves both specificity and control of delivery. Recent work has found that exosome-liposome hybrids were able to successfully deliver CRISPR-Cas9 systems *in vitro* and *in vivo* (Lin et al., 2018; Tao et al., 2018). This is particularly exciting for the future of precision medicine. Importantly, exosomal studies are not without challenge. There is a high degree of heterogeneity in vesicles, variability between *in vitro* and *in vivo* findings, and difficulty in determining vesicle origin or destination. Advances in isolating and characterizing extracellular vesicle-associated lncRNAs will significantly help move the field forward and has the potential to revolutionize clinical medicine.

Notably, even once we are able to identify “druggable” lncRNAs, it is still unclear what the downstream or off-target effects would be and if they would be adverse. Until clinical trials are conducted, the safety and efficacy of lncRNAs as therapeutic targets remains unknown. Evidently, the emerging study of lncRNAs has many challenges, but recent work underscores the importance of the contribution of lncRNAs to the regulation of angiogenesis in health and disease.

## Conclusion

Our review is an overview of angiogenic long non-coding RNAs, and their epigenetic regulation of the vascular endothelium. The functional properties of the vascular endothelium are diverse and heterogeneous between vascular beds. Our understanding of angiogenesis to date has largely focused on protein signaling, but recent work by scientists has revealed that long non-coding RNAs, which are a functionally diverse class of molecules, are involved in regulating this process. As the function of lncRNAs is often dependent on their subcellular localization, nuclear lncRNAs act as epigenetic modifiers. Scientists have begun to identify and characterize a sub-class of lncRNAs: angiogenic lncRNAs. This includes: STEEL, GATA6-AS, and MANTIS, and the disease-associated angiogenic lncRNAs: MEG3, MALAT1, or ANRIL. Taken together, these emerging concepts may provide a novel avenue for therapeutic targets or biomarkers for disease in the next decade.

## Author Contributions

NS, RN, and PAM drafted and edited the manuscript. All authors read and approved the manuscript.

## Conflict of Interest

The authors declare that the research was conducted in the absence of any commercial or financial relationships that could be construed as a potential conflict of interest.

## Publisher’s Note

All claims expressed in this article are solely those of the authors and do not necessarily represent those of their affiliated organizations, or those of the publisher, the editors and the reviewers. Any product that may be evaluated in this article, or claim that may be made by its manufacturer, is not guaranteed or endorsed by the publisher.
